# Supplementary material for: Methods to Assess Fat Mass in Infants and Young Children: A Comparative Study Using Skinfold Thickness and Air-Displacement Plethysmography
Source: Life (Basel). 2021 Jan 20;11(2):75. doi: 10.3390/life11020075 (PMC7909249; doi:10.3390/life11020075)
Supplement: Supplementary file 1 [file life-11-00075-s001.pdf]

life MDPI

*Supplementary Materials:*

# Methods to Assess Fat Mass in Infants and Young Children: A Comparative Study Using Skinfold Thickness and Air-Displacement Plethysmography

**Table S1.** Agreement between the different methods for the prediction of body composition per study centre.

| | 1 Month | 4 Months | 6 Months | 2 Years |
|---|---|---|---|---|
| ADP and skinfold thickness, study centre [a] | | | | |
| n | 138 | 143 | 142 | 72 |
| Mean difference (bias) | –3.01 | 2.41 | 3.23 | 0.13 |
| Limits of agreement (CI95%) | 2.81 | 9.61 | 10.94 | 14.33 |
| | –8.82 | –4.79 | –4.48 | –14.08 |
| Difference [a] | –3.15 | 2.40 | 3.23 | –0.09 |
| *P* [a] | <0.001 | <0.001 | <0.001 | 0.858 |
| (CI95%) | (–3.91, –2.39) | (1.64, 3.16) | (2.47, 3.99) | (–1.07, 0.89) |
| ICC [b] | 0.38 | 0.42 | 0.38 | 0.33 |
| ADP and skinfold thickness, study centre [b] | | | | |
| n | 87 | 86 | 77 | 30 |
| Mean difference | –3.12 | 0.52 | 1.57 | –1.90 |
| Limits of agreement (CI95%) | 3.58 | 7.02 | 8.55 | 10.49 |
| | –9.82 | –5.98 | –5.41 | –14.30 |
| Difference [a] | –3.13 | 0.54 | 1.57 | –2.03 |
| *P* [a] | <0.001 | 0.151 | <0.001 | 0.008 |
| (CI95%) | (–4.10, –2.16) | (–0.43, 1.51) | (0.56, 2.58) | (–3.54, –0.53) |
| ICC [b] | 0.46 | 0.60 | 0.52 | 0.34 |

[a] Differeces between the methods by linear mixed–models analysis, [b] ICC; Intra–class Correlation Coefficient

**Table S2.** Agreement between the different methods for the prediction of body composition per sex.

| | 1 Month | 4 Months | 6 Months | 2 Years |
|---|---|---|---|---|
| ADP and skinfold thickness, boys | | | | |
| n | 113 | 112 | 111 | 82 |
| Difference [a] | –3.40 | 0.70 | 1.78 | –0.57 |
| *P* [a] | <0.001 | 0.112 | <0.001 | 0.341 |
| (CI95%) | (–4.27, –2.53) | (–0.16, 1.57) | (0.90, 2.67) | (–1.74, 0.60) |
| ADP and skinfold thickness, girls | | | | |
| n | 132 | 126 | 124 | 91 |
| Difference [a] | –2.92 | 2.62 | 3.36 | –0.77 |
| *P* [a] | <0.001 | <0.001 | <0.001 | 0.184 |
| (CI95%) | (–3.74, –2.10) | (1.80, 3.45) | (2.54, 4.20) | (–1.92, 0.37) |

[a] Differeces between the methods by linear mixed–models analysis
